# A Rare Complication of an Ingested Foreign Body: Gallbladder Perforation

**DOI:** 10.1155/2013/672572

**Published:** 2013-07-30

**Authors:** Safak Karacay, Koray Topçu, Selami Sözübir

**Affiliations:** ^1^Department of Pediatric Surgery, Liv Hospital Ulus, Bahcesehir University, Beşiktaş, 34340 Istanbul, Turkey; ^2^Pediatric Surgery Clinic, Darica Farabi State Hospital, Kocaeli, Turkey; ^3^Department of Pediatric Surgery and Pediatric Urology, Yeditepe University Faculty of Medicine, Istanbul, Turkey

## Abstract

We present a 13-year-old child who admitted with a dull right upper quadrant pain that started 3 weeks before her referral. Several medications were given but they did not change the intensity and the frequency of the pain. Her physical examination was nonspecific except for slight right upper quadrant tenderness. The imaging studies revealed a sewing pin perforating the stomach and gallbladder. The patient was treated with a successful operation, and no postoperative complications were observed. To our knowledge, this is the first case of a sharp foreign body gallbladder perforation in a child.

## 1. Introduction

Ingestion of foreign bodies (FB) is a common problem during childhood, alerting the family but having relatively low complication rates. While most of these objects leave the alimentary tract without a complicating course, sharp objects may perforate the viscera and present as acute abdomen [1, 2]. We report a 13-year-old girl presenting with a persistent dull abdominal pain. The child remembered that she has swallowed a pin about three weeks prior to her admission after the demonstration of a radio-opaque FB in abdominal X-ray. The report differs from the others with its presentation, which is not an acute abdomen, and presence of gallbladder perforation after piercing the stomach. As far as we know, despite a case in an adult, this is the only child case reported in the literature.

## 2. Case

A 13-year-old girl was admitted to our clinic for intermittent blunt abdominal pain presenting mostly after the meals. The pain was consistent and did not relieve with medications. The physical examination was notable only for slight right upper quadrant (RUQ) tenderness. The biochemical and hematological values were within normal limits. On abdominal X-ray, a radio opaque image was seen. With the deepened history, we learned that the patient has swallowed a sewing pin about three weeks prior to her admission. She was interned due to the location and long stay of the pin for further evaluation. An abdominal ultrasound revealed a 38 mm FB perforating the stomach from antrum and lying over the gallbladder of its superior pole. The 30 mm part of the pin was outside the stomach, and gallbladder wall was edematous (13 mm). 

**Figure 1:**
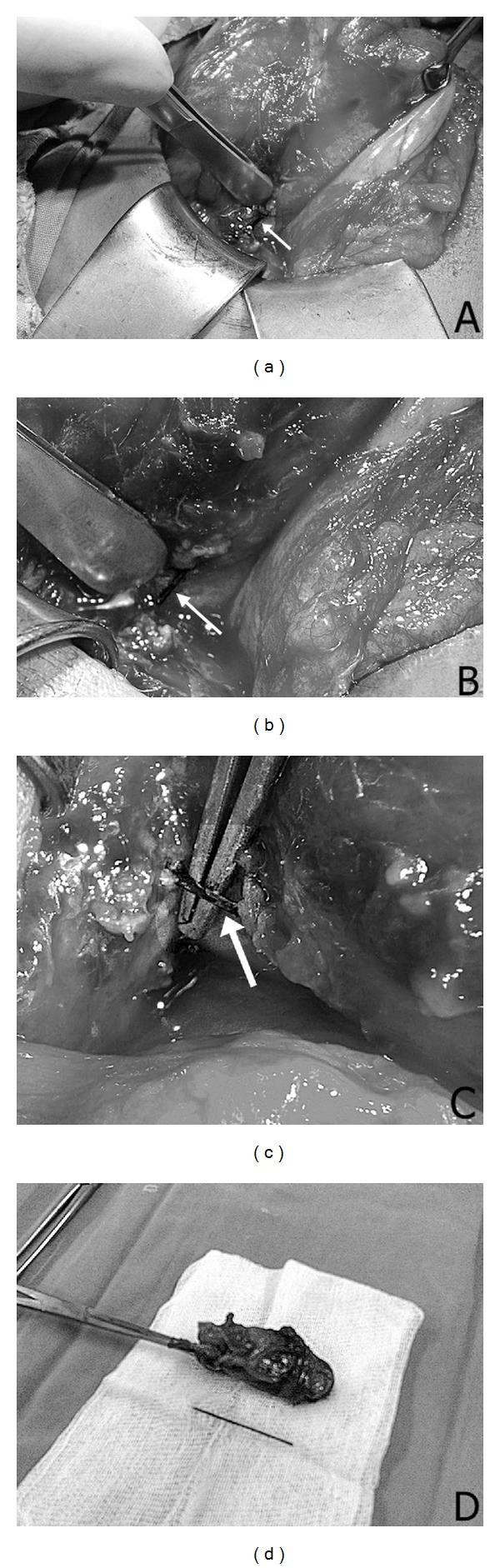
(a) The inflamed area exposed after the retraction of the stomach. (b) Close up revealed oozing of serous fluids between edematous tissues; a small portion of the foreign body is observed. (c) A needle is exposed between the stomach wall and gallbladder. (Arrows pointing at the sewing needle in (a)–(c)). (d) Gallbladder after the cholecystectomy, marked inflammation with thickened wall.

We planned and performed laparotomy on the same day. During the exploration, stomach and the surrounding tissues were observed to be adherent to each other with marked tissue edema, and serous fluid was oozing from the area (Figures 1(a)-1(b)). Dissection over the inflamed area revealed a sewing pin perforating the stomach from the anteromedial part of the small curvature and the gallbladder (Figure 1(c)). The gallbladder was markedly edematous, and the contents were purulent. We repaired the perforation and performed cholecystectomy (Figure 1(d)). The oral feeding was started at the postoperative 3rd day, and we sent the patient home with antibiotics and anti-inflammatory medication the next day. We did not observe any problems during the early or late postoperative period.

## 3. Discussion

Although the ingested FBs are common and alerting problems of childhood, most of them pass the alimentary tract without any sequel. Sharp materials such as fish bones, chicken bones, and needles in contrast may cause complications up to 35% of the cases, such as appendicitis, irreducible inguinal hernia, intussusception, pancreatitis, hepatic abscess, and duodenocolic fistula [1, 2]. 

During the transit through the alimentary tract, foreign bodies can migrate to any abdominal organ, but perforation of the stomach and migration to the gallbladder is extremely rare. Only one case of fishbone in a 67-year-old man was observed in the gallbladder without any fistula in the stomach. The case was managed with laparoscopy, and cholecystectomy was performed [3]. 

In a successful review of Santos et al. in 2007, concerning the adult cases of gastric perforation and hepatic abscess formation, 40 patients were identified [4]. Of these, 4 were autopsy findings, piercing objects leading to the septic shock and death of the patients. Most commonly identified objects were toothpicks and chicken and fish bones. The most common site of perforation was stomach (*n* = 22) while the remaining were duodenum, transverse colon, sigmoid, and appendix. The most common symptoms were found to be epigastralgia, vomiting, fever, nausea, and jaundice. The suffering period of the patients differed from 1 day (toothpick from duodenum to the both lobes of the liver) to 1 year (a needle in the right lobe of the liver).

The perforation of the luminal organs of the abdomen mostly ends up with peritonitis and acute abdomen, necessitating the urgent surgical intervention. A delay in the diagnosis of such a condition results in sepsis and death. In minority of the cases, such as ours, the body isolates the pathology, enclosing the area with organ walls and omentum, creating an encapsulated zone of inflammation. 

The management of gastrointestinal FBs still bears debates. In a guideline for the management of ingested FBs and food impactions reported in 2011, Ikenberry et al. described a myriad of swallowed sharp-pointed objects. Chicken and fish bones, straightened paperclips, toothpicks, needles, bread bag clips, and dental bridgework ingestions have been associated with complications [5–11]. It is stated that sharp-pointed objects, which have passed into the stomach or proximal duodenum, should be retrieved endoscopically if safe removal is possible. Otherwise, followup with daily radiographs for the documentation of the passage is stated to be appropriate [3]. It is also stated that patients with a known history of FB ingestion should be instructed to report abdominal pain, vomiting, persistent temperature elevations, hematemesis, or melena.

A detailed history and wisely planned imaging are the crucial steps in FB ingestion in children. As presented in our case, the child may have forgotten or be frightened and trying to hide or may even be unaware of the condition. Any disturbing examination findings accompanying a chronic abdominal discomfort should lead the surgeon to imaging modalities in a stepwise fashion, starting from X-rays. 

Although we preferred open surgery, laparoscopic approach may also be encouraged in acute and less inflamed cases because it enables direct assessment of the intraperitoneal cavity and organs.
